# Aurora-B kinase pathway controls the lateral to end-on conversion of kinetochore-microtubule attachments in human cells

**DOI:** 10.1038/s41467-017-00209-z

**Published:** 2017-07-28

**Authors:** Roshan L. Shrestha, Duccio Conti, Naoka Tamura, Dominique Braun, Revathy A. Ramalingam, Konstanty Cieslinski, Jonas Ries, Viji M. Draviam

**Affiliations:** 1 Department of Genetics, University of Cambridge, Cambridge, CB2 3EH UK; 2 School of Biological and Chemical Sciences, Queen Mary University of London, London, E1 4NS UK; 3 European Molecular Biology Laboratory, Cell Biology and Biophysics Unit, Meyerhofstrasse 1, Heidelberg, Germany; 4 Present Address: Center for Cancer Research, National Cancer Institute, National Institutes of Health, Bethesda, Maryland 20892 USA; 5 Present Address: Barts Cancer Institute, Queen Mary University of London, London, EC1M 6BQ UK

## Abstract

Human chromosomes are captured along microtubule walls (lateral attachment) and then tethered to microtubule-ends (end-on attachment) through a multi-step end-on conversion process. Upstream regulators that orchestrate this remarkable change in the plane of kinetochore-microtubule attachment in human cells are not known. By tracking kinetochore movements and using kinetochore markers specific to attachment status, we reveal a spatially defined role for Aurora-B kinase in retarding the end-on conversion process. To understand how Aurora-B activity is counteracted, we compare the roles of two outer-kinetochore bound phosphatases and find that BubR1-associated PP2A, unlike KNL1-associated PP1, plays a significant role in end-on conversion. Finally, we uncover a novel role for Aurora-B regulated Astrin-SKAP complex in ensuring the correct plane of kinetochore-microtubule attachment. Thus, we identify Aurora-B as a key upstream regulator of end-on conversion in human cells and establish a late role for Astrin-SKAP complex in the end-on conversion process.

## Abstract (web summary)

Human chromosomes are captured along microtubule walls and then tethered to microtubule-ends through a multi-step end-on conversion process. Here the authors show that Aurora-B regulates end-on conversion in human cells and establish a late role for Astrin-SKAP complex in the end-on conversion process.

## Introduction

During cell division, accurate segregation of DNA requires the proper attachment of chromosomes to microtubules. Chromosome-microtubule attachment relies on a macromolecular structure—the kinetochore—that assembles on the centromeric region of chromosomes. We and others showed that kinetochores are predominantly captured along the walls of microtubules (termed lateral kinetochores) and then tethered onto the ends of microtubules (termed end-on kinetochores)^1–4^. This dramatic change in the geometry of kinetochore-microtubule (KT-MT) interaction is achieved through a multi-step end-on conversion process. End-on conversion is an indispensable process for lateral kinetochores: only when the ends of microtubules are tethered to the kinetochore, the growth and shrinkage of microtubule-ends (K-fibres) can impart pushing or pulling forces on the chromosome^5–7^. Lesions in the end-on conversion process lead to defective chromosome segregation, as seen in cells lacking the loop region of the kinetochore protein HEC1/Ndc80^4, 8–13^, highlighting the importance of understanding how a lateral kinetochore is converted into an end-on kinetochore.

Several evolutionarily conserved kinetochore proteins are known to be important for forming mature attachments capable of load-bearing and end-on pulling events^2–4, 8, 14–16^. Using deconvolution microscopy, we recently reported two markers to distinguish the plane of KT-MT attachment in human cells: (i) Mature end-on kinetochores, but not lateral kinetochores, recruit the Astrin-SKAP complex (ii) Mature end-on kinetochores, but not lateral kinetochores, are capable of converting the changes in K-fibre length into kinetochore movements^4^. However, upstream signaling pathways that control the end-on conversion process have not been established so far in human cells. In yeasts, Aurora-B (Ipl1) kinase was shown to be an important upstream regulator of the end-on conversion process^17^. Whether Aurora-B plays a similar role in regulating the end-on conversion process in human cells is not known.

Distinct from the end-on conversion process that ensures the correct plane of KT-MT attachment, the error correction process ensures the correct orientation of attachment (referred as biorientation; reviewed in ref. ^9^). Biorientation defects are resolved by Aurora-B kinase enriched at centromeres through feedback loops^18–20^; it phosphorylates outer-kinetochore substrates causing the detachment of non-bioriented KT-MT attachments (e.g., syntelic end-on attachments)^16, 21–27^. In addition, active Aurora-B has been reported in human kinetochores during early mitosis^28^ and specifically on kinetochores that are laterally attached^29^. Whether Aurora-B at the outer-kinetochore would destabilise immature lateral attachments is however not known.

Aurora-B and its counteracting phosphatases, PP1 and PP2A, are important for regulating outer-kinetochore assembly, KT-MT attachment stability, chromosome alignment and checkpoint function^29–38^. Several Aurora-B counteracting phosphatases are recruited to the centromere and kinetochore in a temporally and spatially restricted manner (reviewed in refs ^39, 40^). Whether Aurora-B counteracting phosphatases play a role in controlling the plane of KT-MT attachment remains unclear.

Here, we examine the role of Aurora-B kinase and its counteracting phosphatases in the end-on conversion process. We report that Aurora-B kinase impacts the end-on conversion process differently dependent on its sub-cellular localization—outer kinetochore vs. centromere. While Aurora-B targeted to the outer-kinetochore detaches lateral kinetochores prior to end-on conversion, Aurora-B targeted to the centromere stabilizes lateral kinetochores and retards end-on conversion. We find that lateral KT-MT attachments are relatively ‘immune’ to Aurora-B. Next, of the two Aurora-B-counteracting phosphatases, we find that BubR1-associated PP2A, but not KNL1-associated PP1, is the most potent regulator of the end-on conversion process. Finally, we identify the Astrin-SKAP complex as a late player in the end-on conversion process. Thus, we report a novel spatially controlled role for Aurora-B in the end-on conversion process, establish BubR1-associated PP2A as a key phosphatase that counteracts Aurora-B activity during end-on conversion and finally, demonstrate a late role for Aurora-B regulated Astrin-SKAP complex in the end-on conversion process. This study provides the first insight into how Aurora-B mediated signaling controls the plane of kinetochore-microtubule attachments in human cells.

## Results

### Aurora-B activity is high on immature lateral kinetochores

**Fig. 1:**
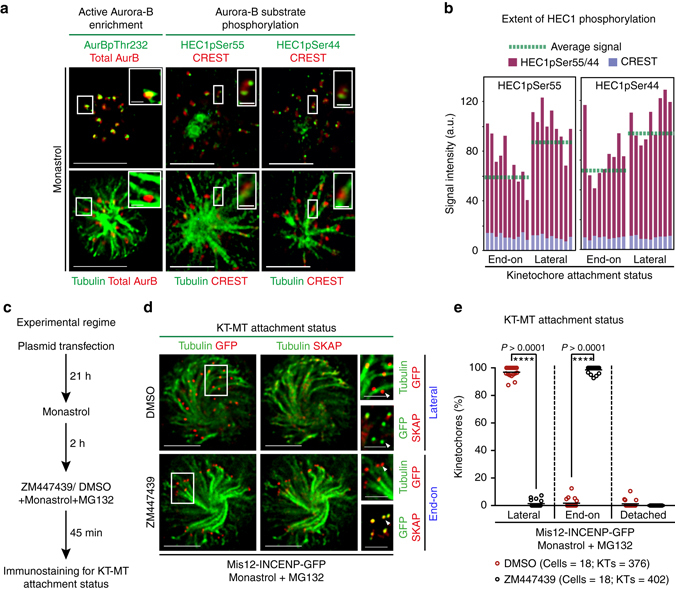
High Aurora-B activity promotes KT attachment to MT-walls. **a** Representative images show high Aurora-B activity on lateral kinetochores. Monastrol treated cells were immunostained with antibodies against Tubulin, Aurora-BpThr232 and total Aurora-B (AurB) (*left panel*) or CREST antisera and antibodies against Tubulin and either HEC1pSer55 (*middle panel*) or HEC1pSer44 (*right panel*). Cropped images show lateral-kinetochores. Scale: 5 μm in uncropped images; 1 μm in cropped images. **b** Graphs show higher average signal intensities of HEC1pSer55 (*left*) and HEC1pSer44 (*right*) in lateral compared to end-on kinetochores as assessed from at least nine randomly chosen kinetochores from cells in **a**. CREST signal intensities are used as internal controls. **c** Experimental regime: Cells transfected with plasmid vectors encoding Mis12-INCENP-GFP were exposed to Monastrol and MG132 with either ZM447439 or DMSO (solvent control), prior to immunostaining. **d** Images of cells expressing Mis12-INCENP-GFP treated as in **c** and immunostained with antibodies against Tubulin, SKAP and GFP. *White arrowheads* in cropped images show ‘Lateral’ kinetochore lacking SKAP (*upper panel*) and ‘End-on’ kinetochore enriched with SKAP (*lower panel*). Scale: 5 μm in uncropped and 2 μm in cropped images. *Boxed areas* in **a** and **d** correspond to cropped images. **e** Graph shows percentage of lateral, end-on and detached kinetochores in Mis12-INCENP-GFP expressing cells treated as in **c**. Each *circle* represents value from one cell. *Black horizontal* bar marks average values from three independent experimental repeats. ‘*’ indicates statistically significant difference on the basis of *P*-values obtained using unpaired Student’s *t*-test

We first quantified and confirmed the presence of active Aurora-B on lateral kinetochores. For this purpose, HeLa cells were exposed to Monastrol to generate monopolar spindles, which mimic an early mitotic spindle configuration and allow clear distinction between lateral kinetochores and end-on kinetochores^4^. Immunostaining with antibodies against activating phosphorylation of Aurora-B (pThr232) (Supplementary Fig. 1A) showed that activated Aurora-B is abundant on lateral kinetochores (*n*_KTs_ > 30; *n*_cells_ = 10; Fig. 1a). Consistent with this finding, immunostaining with phospho-specific antibodies against Aurora-B substrate sites of the outer KT protein HEC1, Ser44 and Ser55^28^, showed higher HEC1 phosphorylation on lateral compared to end-on kinetochores (*n*_KTs_ > 20; *n*_cells_ = 10; Fig. 1a,b). Thus, Aurora-B activity is high on lateral kinetochores.

### Aurora-B at outer-kinetochore facilitate lateral kinetochores

To determine the contribution of outer-kinetochore associated Aurora-B activity in early mitosis, we locally increased Aurora-B activity at the outer-kinetochore by expressing the Mis12-INCENP-GFP fusion protein, wherein a fragment of INCENP that lacks the centromere targeting domain but retains the Aurora-B docking domain is fused to the outer-kinetochore protein Mis12^26^. As expected, but not reported so far, Mis12-INCENP-GFP expressing HeLa cells showed high levels of HEC1pSer44 phosphorylation at all kinetochores (Supplementary Fig. 1B), confirming the fusion protein’s ability to retain Aurora-B activity at the outer kinetochore. Hence, this approach allowed us to quantify the effect of a localised increase in Aurora-B activity.

To study KT-MT attachment status (end-on vs. lateral) during early mitosis when end-on conversion events occur, we immunostained Monastrol-treated Mis12-INCENP-GFP expressing cells with antibodies against GFP, Tubulin and SKAP, a microtubule-end binding protein^41, 42^ specifically enriched on end-on but not lateral kinetochores^4^ (Fig. 1c). In monopolar spindles of control cells, less than 25% of kinetochores were laterally attached and they lacked SKAP^4^. However, in Mis12-INCENP-GFP expressing cells, more than 95% of kinetochores were laterally attached (Fig. 1d,e) and they lacked SKAP (*n*_cells_ = 26/31) (Fig. 1d and Supplementary Fig. 1C). The striking defect in the plane of KT-MT attachment observed in Mis12-INCENP-GFP expressing cells was completely reversed following Aurora-B inhibition using ZM447439, as almost all kinetochores were attached to MT-ends and enriched for SKAP (*n*_cells_ = 20/20) (Fig. 1d,e and Supplementary Fig. 1C). Thus, retaining Aurora-B activity at the outer-kinetochore promotes the incidence of lateral kinetochores, suggesting a failure in the end-on conversion process. The inhibitor studies confirm that this failure is not due to non-specific interference by Mis12-INCENP-GFP fusion protein, but is caused by Aurora-B activity. We conclude that outer-kinetochore associated Aurora-B activity increases the incidence of lateral kinetochores in monopolar spindles that mimic an early mitotic spindle configuration.

### Congression resolves Aurora-B induced lateral attachments

We tested if constitutive Aurora-B activity would increase the incidence of lateral kinetochores in bipolar spindles that allow biorientation and inter-kinetochore tension. To study bipolar spindles, we treated cells with MG132, a proteasome inhibitor. MG132 treated cells expressing Mis12-INCENP-GFP displayed a mixture of both congressed and uncongressed kinetochores (Supplementary Fig. 2A–C), as previously reported^26^. In cells with congressed KTs, we observed KT-MT attachments that were properly bioriented (Supplementary Fig. 2A, insets) and predominantly end-on attached, with a small but statistically significant fraction displaying lateral attachments (Supplementary Fig. 2D). Thus, bipolar spindles partially rescue the end-on conversion defect induced by constitutive Aurora-B activity. We confirmed this finding by quantifying Astrin-SKAP recruitment at kinetochores: although the intensity of KT-associated SKAP was reduced by ~2.5-fold in Mis12-INCENP-GFP expressing cells compared to non-expressing cells, in agreement with previous report^43^ (Supplementary Fig. 2E and F), SKAP was clearly enriched on congressed and bioriented kinetochore pairs. Thus, bipolar spindles, which allow chromosome congression and biorientation of KT-MT attachments, are able to overcome the end-on conversion defect induced by Aurora-B at the outer-kinetochore.

### Aurora-B at outer-kinetochore abrogates end-on conversion

**Fig. 2:**
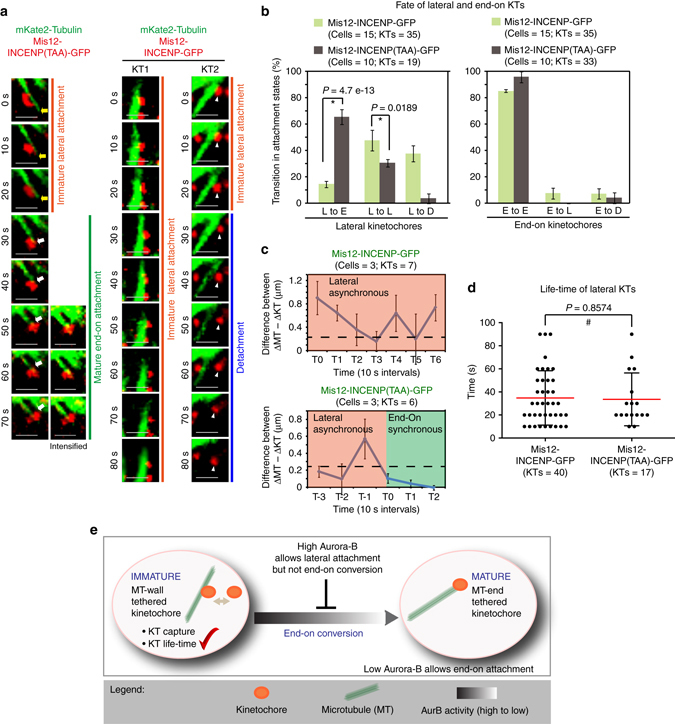
Constitutive Aurora-B activity at the outer-kinetochore allows lateral attachments but disrupts end-on conversion. **a** Single-plane time-lapse images of Z-stacks show the fate of a lateral KT (*red*) attached to MT (*green*). Time-lapse images of HeLa cells expressing mKate2-Tubulin and either Mis12-INCENP-GFP or Mis12-INCENP (TAA)-GFP mutant were acquired in the presence of Monastrol. *Yellow arrows* mark shrinking lateral K-fibre and *white arrows* show the synchronous movement of the KT with MT-end. *White arrowheads mark* the KT tracked. For three final time-points (*left panels*) ‘intensified’ images are included to highlight the K-fibre. Scale bar: 2 μm. **b** Graphs show the percentage of lateral (*left*) or end-on (*right*) kinetochores that transitioned into other attachment states (D-detached, L-lateral and E-end-on) in time-lapse movies as in **a**. **c** Graph shows the difference between ΔMT (change in MT-end position) and ΔKT (change in KT position) through time, used to define synchronous or asynchronous movements. Values less than 0.25 μm (marked by *dashed line*), sustained for at least 30 s, are indicated as synchronous (*green*) movement between the KT and MT-end. All other values are indicated as asynchronous movements of the KT and MT-end. **d** Graph shows the time spent by lateral kinetochores on MT-walls, before changing into another attachment state, in cells expressing either Mis12-INCENP-GFP or Mis12-INCENP (TAA)-GFP. *Red* and *black* bars mark mean-time and SD values, respectively, across kinetochores in three experiments. Error bars in **b** and **c** are SEM values across experiments **b** or across kinetochores **c**. ‘*’ and ‘#’ indicate statistically significant and insignificant differences, respectively, on the basis of *P*-values obtained across three experimental repeats using a Proportion test **b** or unpaired Student’s *t*-test **d**. **e** Cartoon shows how Aurora-B at the outer-kinetochore disrupts the end-on conversion process by detaching lateral kinetochores prior to end-on conversion. Aurora-B at the outer-kinetochore does not interfere with the capture or maintenance of kinetochores on microtubule walls. Reducing Aurora-B activity allows the formation of mature end-on attachments

To understand the precise reason for the accumulation of lateral attachments in Mis12-INCENP-GFP expressing cells, we investigated three possibilities: (i) whether lateral kinetochores are due to excessive error-correction (i.e., detachment of end-on kinetochores promoting their recapture onto microtubule walls), (ii) abrogation of the process of converting lateral attachments into end-on attachments or (iii) excessive stabilization of lateral KT-MT interactions. To distinguish between all three mechanisms, we performed time-lapse microscopy of monopolar spindles of cells co-expressing mKate2-Tubulin and Mis12-INCENP-GFP. We tracked the fate of lateral and end-on kinetochores and analysed changes in the plane of KT-MT interaction and KT movements. A Mis12-INCENP(TAA)-GFP mutant with compromised Aurora-B activity^26, 44–46^ was used as a negative control. In control cells expressing Mis12-INCENP(TAA)-GFP, lateral kinetochores bound to microtubule walls were converted into end-on kinetochores as expected (Fig. 2a). Quantitative analysis of the fate of kinetochores in these control cells showed that the vast majority of lateral kinetochores became end-tethered within 50 s (Fig. 2b), following which they underwent forward and regressing movements, which were synchronous with changes in K-fibre length (Fig. 2a (*left panels*) and c (*lower panel*)). Such synchronous movement between the kinetochore and MT-end demonstrates the establishment of a productive and mature KT-MT attachment that are capable of converting the change in K-fibre length into kinetochore movement. In stark contrast, in cells expressing Mis12-INCENP-GFP, majority of lateral kinetochores failed to convert into end-on kinetochores (Fig. 2b). In these cells, kinetochore movements were not synchronous with changes in K-fibre length (Fig. 2a (*right panels*) and c (*upper panel*)), demonstrating an immature KT-MT attachment state. A significant proportion of lateral kinetochores in Mis12-INCENP-GFP expressing cells underwent detachment (Fig. 2a (*right panels*) and Fig. 2b (*left panel*)) and reattached onto microtubule walls (Fig. 2a (*right panels*)). However, the lifetime of lateral kinetochores was not significantly different in Mis12-INCENP-GFP and Mis12-INCENP(TAA)-GFP expressing cells (Fig. 2d). This unaltered life-time of lateral kinetochores shows that Aurora-B at the outer-kinetochore does not prohibit the capture or the maintenance of lateral kinetochores. Based on our data on KT-MT attachment status, KT movements and KT life-time in live-cells, we conclude that Aurora-B activity allows kinetochore capture and maintenance along microtubule walls but it specifically disrupts the end-on conversion process.

Finally, as KT fate analysis showed that end-on (monotelic) kinetochores remained tethered to MT-ends and did not detach in Mis12-INCENP-GFP expressing cells (Fig. 2b, *right panel*), we are able to rule out excessive error-correction (detachment of end-on kinetochores) as an indirect cause for increase in lateral kinetochores. In summary, we reveal a novel role for Aurora-B in selectively abrogating the end-on conversion process, without disrupting the establishment or maintenance of lateral attachments (Fig. 2e). We conclude that this novel role of Aurora-B in influencing the plane of KT-MT attachment in human cells is independent of the kinase’s established role in the error-correction process.

### Distinct Aurora-B pools disrupt end-on conversion differently

**Fig. 3:**
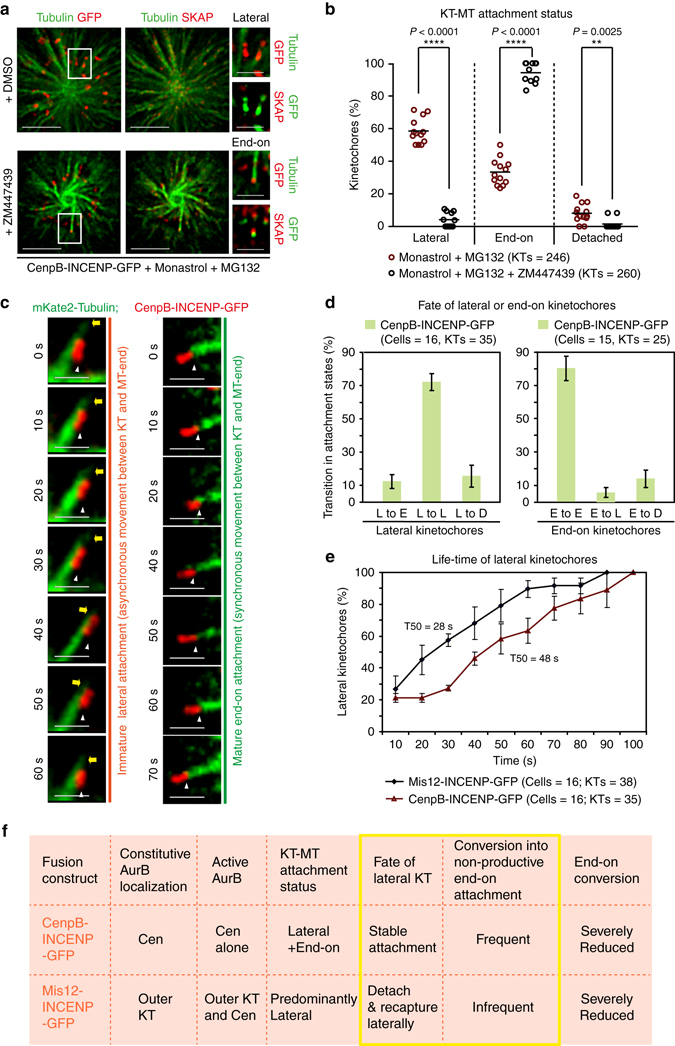
Spatially distinct pools of Aurora-B impede distinct steps of the end-on conversion process. **a** Images of cells expressing CenpB-INCENP-GFP exposed to Monastrol and MG132 with either ZM447439 or DMSO (solvent control), as indicated, prior to immunostaining with antibodies against Tubulin, SKAP and GFP. Cropped images show lateral (*upper row*) and end-on (*lower row*) kinetochores. Scale: 5 μm in main and 2 μm in cropped images. *Boxed areas* correspond to cropped images. **b** Graph shows percentage of lateral, end-on and detached kinetochores in CenpB-INCENP-GFP expressing cells treated as in **a**. Each *circle* represents values from one cell. *Black bar* marks average values from two independent experimental repeats. * indicates statistically significant difference on the basis of *P*-values obtained using unpaired Student’s *t*-test. **c** Single-plane time-lapse images of Z-stacks show the fate of a lateral or end-on KT (*red*) bound to MT (*green*). Time-lapse images of HeLa cells expressing mKate2-Tubulin and CenpB-INCENP-GFP treated with Monastrol. *White arrowheads* mark KT tracked. *Yellow arrows* mark the tip of lateral K-fibre. Scale: 2 μm. **d** Graph shows percentage of lateral or end-on kinetochores that transitioned into other attachment states (D-detached, L-lateral and E-end-on) in time-lapse movies as in **c**. Error bars are SEM values across three experimental repeats **e** Cumulative frequency plots show time spent by lateral kinetochores on MT-walls, before changing into another attachment state, in cells expressing either Mis12-INCENP-GFP or CenpB-INCENP-GFP. Error bars are SEM values across three experimental repeats. Note: Mis12-INCENP-GFP curve values were obtained from data presented in Fig. 3d. T50 values are in seconds derived from the cumulative frequency plot. **f** Table contrasts the consequence of localizing Aurora-B at the outer kinetochore (KT) vs. centromere (cen), using Mis12-INCENP-GFP and CenpB-INCENP-GFP fusion proteins, respectively. Both fusion proteins disrupt end-on conversion but they differently control the fate of lateral attachments (within *yellow box*). Active Aurora-B status (Active AurB) was assessed using immuno-staining with antibodies against AuroraB-pThr232 as in Fig. 1a. KT-MT attachment status was obtained from fixed-cell studies. Fate of lateral kinetochore and instances of non-productive end-on attachments and productive end-on conversion are from live-cell movies

Because active Aurora-B is lost from outer-kinetochore and retained only at the inner centromeric region during metaphase^28^, we tested whether centromere localized Aurora-B influences the end-on conversion process similarly to outer-kinetochore localized Aurora-B. For this purpose, we performed fixed and live-cell microscopy studies using CenpB-INCENP-GFP, wherein the Aurora-B docking domain is fused to a centromeric protein, CenpB^26^. As expected CenpB-INCENP-GFP signals were predominantly centromeric and did not extent beyond inner kinetochore region (Supplementary Fig. 5B). In monopolar spindles of CenpB-INCENP-GFP expressing cells, nearly 60% of kinetochores were laterally attached and they lacked SKAP of the Astrin-SKAP complex (Fig. 3a,b), suggesting an end-on conversion defect. This lateral attachment phenotype and lack of Astrin-SKAP complex was rescued under two conditions: in monopolar spindles treated with Aurora-B inhibitor (Fig. 3a,b) and in bipolar spindles with congressed chromosomes (Supplementary Fig. 3A and B). We conclude that a local increase in Aurora-B, restricted to centromere and inner kinetochore region, increases the incidence of lateral kinetochores in monopolar spindles and this altered plane of KT-MT interaction is rescued in congressed kinetochores of bipolar spindles.

To determine the impact of centromere bound Aurora-B on lateral kinetochores, we tracked the fate of kinetochores in cells co-expressing mKate2-Tubulin along with either CenpB-INCENP-GFP or CenpB-INCENP(TAA)-GFP (negative control^26^). In control cells expressing CenpB-INCENP(TAA)-GFP, the majority of kinetochores were attached to microtubule-ends and stably tethered without detachment episodes (Supplementary Fig. 4A and B). As expected, lateral kinetochores that matured into end-on kinetochores underwent forward and regressing movements, synchronous with changes in the K-fibre length (Supplementary Fig. 4C). In stark contrast, cells expressing CenpB-INCENP-GFP displayed a low incidence of lateral to end-on conversion events (Fig. 3c,d). Detachment episodes of lateral or end-on kinetochores were not high (Fig. 3c,d; while 4/4 syntelic KTs detached, only 3/21 monotelic KTs detached within 10 s). Quantitative analysis of kinetochore movements in CenpB-INCENP-GFP expressing cells showed that lateral kinetochores remained laterally attached and failed to sustain synchronous movements with the MT-ends of the K-fibre (Fig. 3d and Supplementary Fig. 4D), revealing a role for centromere bound Aurora-B in retarding the end-on conversion event.

While both centromeric and outer-kinetochore pools of Aurora-B disrupt end-on conversion, there were two key differences: (i) the lifetime of lateral kinetochores was significantly increased in cells expressing CenpB-INCENP-GFP compared to Mis12-INCENP-GFP (Fig. 3e) and (ii) the number of lateral kinetochores that formed non-productive end-on attachments (end-on attachments lasting less than 30 s) was at least twofolds more in cells expressing CenpB-INCENP-GFP (*n*_KT_ = 13/34) compared to Mis12-INCENP-GFP (*n*_KT_ = 6/37).

In summary, we conclude that a local increase of Aurora-B at the outer-kinetochore detaches lateral attachments prior to end-on conversion, whereas Aurora-B at the centromere retains lateral attachments and prevents stable end-on conversion (Fig. 3f). This difference could not be simply explained by gross differences in the extent of fusion protein expression at the kinetochore (Supplementary Fig. 5A) or extent of HEC1 phosphorylation (Supplementary Fig. 5B and C). We propose that the two different spatial pools of Aurora-B may influence downstream targets differently or to different extent leading to distinct impacts upon the end-on conversion process.

### BubR1-PP2A but not KNL1-PP1 controls end-on conversion

**Fig. 4:**
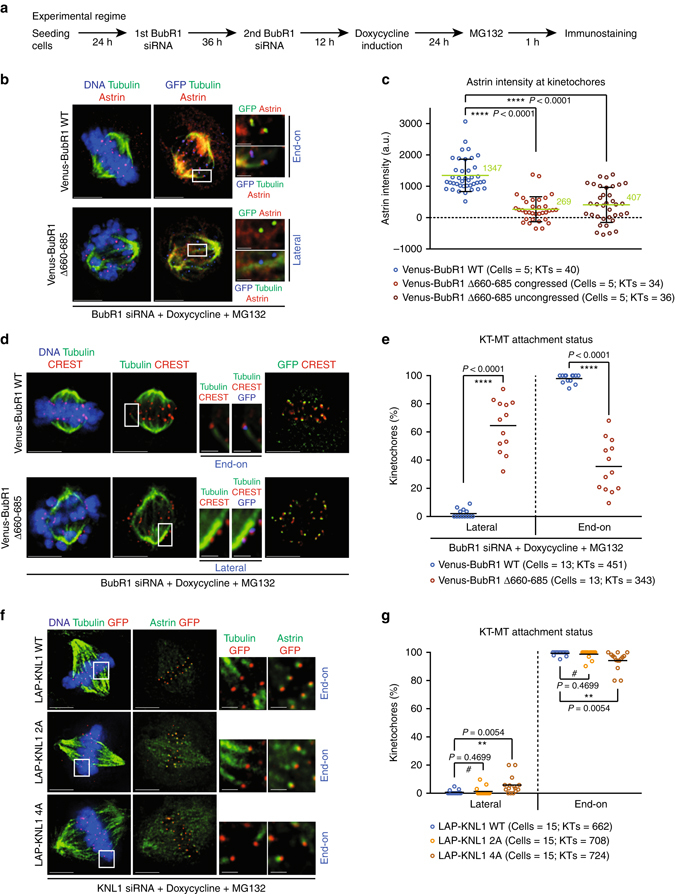
BubR1 associated PP2A, but not KNL1 associated PP1, plays a significant role in the end-on conversion process. **a** Experimental regime: BubR1 siRNA treated HeLa FRT/TO cells conditionally expressing Venus (YFP)-BubR1 (WT or ∆660–685 mutant) were exposed to Doxycycline for 24 h and MG132 for 1 h prior to immunostaining. **b** Images of cells treated as in **a**, immunostained with antibodies against GFP, Tubulin and Astrin and stained with DAPI for DNA. Scale: 5 µm in uncropped and 1 µm in cropped images. Boxed areas correspond to cropped images. **c** Graph of Astrin intensities on congressed or uncongressed kinetochores in cells expressing Venus (YFP) tagged -BubR1 WT or ∆660–685 mutant, as in **b**. *Horizontal lines* show average values (in *green*) across KTs from two independent experiments. Each *circle* represents values from one kinetochore. **d** Images of cells treated as in **a**, immunostained with antibodies against GFP and Tubulin and CREST anti-sera and stained with DAPI for DNA. Scale: 5 µm in uncropped and 1 µm in cropped images. *Boxed areas* correspond to cropped images. **e** Graph shows percentage of lateral vs. end-on kinetochores in Venus-BubR1 (WT or ∆660–685 mutant) expressing cells treated as in **d**. Each *circle* represents values from one cell. *Black bar* marks average values from four independent experiments. **f** Images of KNL1 siRNA treated HeLa cells expressing LAP-tagged KNL1 WT or mutants (2A or 4A). Following plasmid transfection, cells were exposed to Doxycycline for 1 h and then incubated in Doxycycline-free media for 30 h. Prior to fixation cells were exposed to MG132 for 45 min and immunostained with antibodies against GFP, Tubulin and Astrin. Scale: 5 µm in uncropped and 2 µm in cropped images. Boxed areas correspond to cropped images. **g** Graph shows percentage of lateral vs. end-on kinetochores in LAP tagged KNL1 (WT, 4A or 2A mutant) expressing cells, treated as in **f**. Each *circle* represents values from one cell. *Black bar* marks average values from three independent experimental repeats. In **c**, **e** and **g**, ‘*’ and # indicate statistically significant and insignificant differences, respectively (assessed using *P*-values from unpaired Student’s *t*-test)

We hypothesized that Aurora-B counteracting phosphatases at the outer-kinetochore should be essential for end-on conversion, as Aurora-B activity is high at the outer-kinetochore during early prometaphase^47^. At the outer-kinetochore, two major phosphatases (KNL1 associated PP1 and BubR1 associated PP2A) counteract Aurora-B activity and are needed for the spindle assembly checkpoint or maintaining cold-stable KT-MT attachments^30–36^. The extent to which the two phosphatases control the end-on conversion process has not been studied so far. To address this, we first quantified KT-MT attachment status (end-on vs. lateral) in cells conditionally expressing a Venus (YFP) tagged BubR1 mutant that can not interact with PP2A-B56 (BubR1 Δ660–685)^31^ (Fig. 4a). Consistent with previous reports^31, 32, 48^, our immunostaining studies showed that in cells depleted of endogenous BubR1, the expression of the BubR1 mutant lacking the PP2A docking domain interfered with both PP2A recruitment and chromosome alignment (Supplementary Fig. 6A, C and E and Fig. 4b). We next assessed the enrichment of Astrin-SKAP complex (a marker of mature end-on attachments) at congressed kinetochores in cells treated with MG132. Cells expressing the BubR1 Δ660–685 mutant failed to recruit Astrin normally in the vast majority of congressed kinetochores, and the kinetochores that displayed Astrin showed a steep fivefold reduction compared to controls (Fig. 4c). Analysis of KT-MT attachment status in MG132-treated BubR1 Δ660–685 mutant expressing cells demonstrated a highly significant increase in laterally attached kinetochores (Fig. 4d,e) in both congressed and uncongressed kinetochores (Supplementary Fig. 7A). Lateral KT-MT attachment status in BubR1 Δ660–685 mutant expressing cells was further confirmed using super-resolution microscopy (Supplementary Fig. 7B). These data indicate an impairment of the end-on conversion process in cells lacking BubR1 associated PP2A, despite chromosome congression. Based on the status of KT-MT attachment and Astrin recruitment, we conclude that (i) laterally attached kinetochores predominate in cells lacking BubR1 associated PP2A and (ii) chromosome congression is insufficient to rescue the end-on conversion defect in cells lacking BubR1 associated PP2A-B56.

We next tested if KNL1 associated PP1 phosphatase is required for end-on conversion by comparing KT-MT attachment changes induced by two LAP-tagged KNL1 mutants: (i) KNL1-2A where Aurora-B mediated phosphorylation sites at the PP1 docking motif are mutated to allow constitutive recruitment of PP1 and (ii) KNL1-4A where the PP1 docking RVSF motif is mutated to abrogate PP1 recruitment^30, 37^. We first confirmed that PP1 levels are reduced in KNL1 depleted cells expressing LAP-tagged KNL1-4A compared to LAP-tagged KNL1-WT or LAP-tagged KNL1-2A (Supplementary Fig. 6B and D), as reported^37^. Following MG132 treatment, KNL1 depleted cells expressing either LAP-tagged KNL1-2A or KNL1-WT displayed kinetochores that were congressed and normally end-tethered; whereas, cells expressing LAP-tagged KNL1-4A mutant displayed a mild congression defect (Fig. 4f,g and Supplementary Fig. 6F). A slight increase in lateral kinetochores and reduction in Astrin intensity were observed in cells expressing LAP tagged KNL1-4A mutant, compared to cells expressing either KNL1-2A mutant or KNL1-WT fusion proteins (Fig. 4g and Supplementary Fig. 6G). However, the percentage of lateral kinetochores and extent of Astrin reduction were much lesser in cells expressing the PP1 docking mutant (KNL1-4A) compared to PP2A docking mutant (BubR1 Δ660–685) (Compare Fig. 4e,g; Fig. 4c and Supplementary Fig. 6G). These studies provide first insight into the extent to which the two phosphatases control the plane of KT-MT interaction and highlight the essential role of BubR1 associated PP2A in controlling the maturation of KT-MT attachments.

### Astrin-SKAP is a target of Aurora-B during end-on conversion

**Fig. 5:**
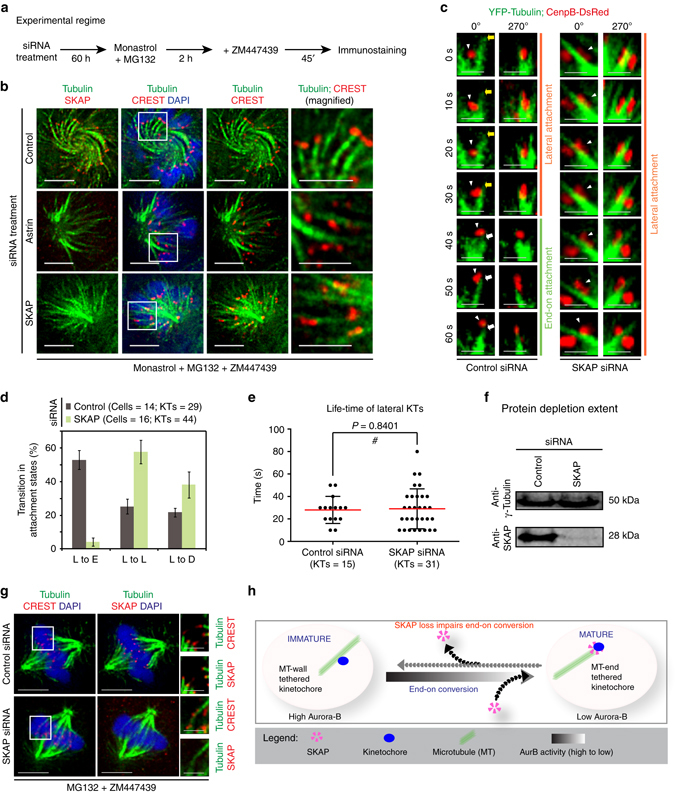
Astrin-SKAP complex mediates a late step of the end-on conversion process. **a** Experimental regime: Cells treated with Monastrol and MG132 for 2 h and ZM447439 for 45 min were immunostained using antibodies against Tubulin and SKAP and CREST antisera. DNA was stained using DAPI. **b** Immunofluorescence images of HeLa cells transfected with control, SKAP or Astrin siRNA, as indicated, and treated as in **a**. Magnified images in the right-most panels correspond to *boxed area*. Scale bar: 5 μm. **c** Single-plane time-lapse images of Z-stacks show the fate of lateral kinetochores (*red*) attached to MTs (*green*) in Control or SKAP siRNA treated HeLa^YFP-Tub; CenpB-Red^ cells in Monastrol. *White arrowheads* mark the KT tracked. *Yellow arrows* mark shrinking lateral K-fibre and *white arrows* highlight synchronous movement of the KT and MT-end. Scale bar: 2 μm. **d** Graph shows percentage of lateral kinetochores that remained lateral (L) or transitioned into other attachment states (D–detached; E–end-on) in time-lapse movies as in **c**. Error bars represent SEM from four experiments. **e** Graph of time spent by lateral kinetochores on MT-walls, before changing into another attachment state, in control or SKAP siRNA treated cells. *Red* and *black* bars mark mean-time and SD values, respectively, across kinetochores from four independent experiments. # indicates statistically insignificant difference (unpaired Student’s *t*-test). **f** Immunoblots of lysates of cells treated with control or SKAP siRNA harvested after time-lapse microscopy. Antibodies against SKAP and γ-Tubulin (loading control) were used. **g** Images of bipolar spindles with congressed chromosomes in control or SKAP siRNA transfected cells treated with MG132 and ZM447439 and immunostained as in **a**. Scale bar: 5 μm in uncropped and 2 μm in cropped images. *Boxed areas* correspond to cropped images. **h** Cartoon illustrates SKAP’s role in the end-on conversion process: (i) SKAP is selectively recruited to end-on (mature) but not lateral (immature) kinetochores. (ii) SKAP is crucial for the lateral to end-on conversion event but not required for tethering kinetochores onto microtubule walls. Finally, reducing Aurora-B allows end-on attachments in a SKAP dependent manner

Because Aurora-B negatively regulates Astrin-SKAP levels at kinetochores^43^, Astrin-SKAP complex is a potential downstream target of Aurora-B in the end-on conversion process. We quantified KT-MT attachment status in monopolar spindles of Aurora-B inhibited cells depleted of either Astrin or SKAP (Fig. 5a). As expected, in control siRNA treated cells, kinetochores were tethered to MT-ends and were uniformly distant from the spindle poles (Fig. 5b). In contrast, in SKAP siRNA treated cells, kinetochores were dispersed along MT-walls and a reduced proportion of the kinetochores were attached to MT-ends (Fig. 5b and Supplementary Fig. 8A), showing a defect in tethering kinetochores to microtubule-ends despite Aurora-B inhibition. A similar phenotype of increased lateral kinetochores was observed in cells treated with Astrin siRNA despite Aurora-B inhibition (Fig. 5b and Supplementary Fig. 8A). We confirmed that SKAP was absent on the kinetochores of Astrin or SKAP siRNA treated cells (Fig. 5b). We conclude that the Astrin-SKAP complex plays a crucial role in ensuring the correct plane of KT-MT interaction.

### KT attachments to MT-ends but not MT-walls rely on SKAP

To determine why end-tethering of kinetochores is compromised in SKAP depleted cells, we tracked the fate of lateral kinetochores in time-lapse movies of cells co-expressing CenpB-DsRed and YFP-Tubulin (Fig. 5c). Analysing the fate of kinetochores showed that in control cells, lateral kinetochores converted into end-on kinetochores normally (Fig. 5d). However, in SKAP depleted cells, lateral kinetochores either remained attached to microtubule walls or detached from microtubule walls and failed to convert into end-on kinetochores (Fig. 5d), showing a failure in the end-on conversion process. The lifetime of lateral kinetochores was comparable in control and SKAP depleted cells (Fig. 5e,f), indicating a dispensable role for SKAP in capturing and maintaining kinetochores along microtubule walls. Although the conversion of a lateral kinetochore into an end-on kinetochore was relatively infrequent in SKAP depleted cells compared to control depleted cells (Fig. 5d), end-tethered kinetochores could be observed in SKAP depleted cells (Supplementary Fig. 8A). Tracking the fate of these end-tethered kinetochores in SKAP depleted cells showed that they rarely remained end-tethered for longer than 30 s, and instead they either became laterally attached or detached (*n*_cells_ = 4; *n*_KTs_ = 12). Thus, SKAP facilitates the end-on conversion event and is essential for the stable maintenance of kinetochores at MT-ends but not MT-walls, demonstrating a late role for SKAP in the end-on conversion process.

To confirm that the defect in the plane of KT-MT attachment observed following SKAP depletion is not an siRNA off-target effect, we used a cell line that conditionally expresses an siRNA resistant form of SKAP fused to GFP (GFP-SKAP^siRes^)^4, 41^. Following SKAP siRNA treatment, monopolar spindles of GFP-SKAP^siRes^ expressing cells exposed to ZM447439 and MG132 (Supplementary Fig. 8B) displayed end-on attached kinetochores, demonstrating a successful rescue of the SKAP depletion induced lateral attachment phenotype (Supplementary Fig. 8C). Thus, we show the first evidence for SKAP’s role in the end-on conversion process, and demonstrate that Aurora-B acts through Astrin-SKAP complex to ultimately define the plane of KT-MT attachment (Fig. 5h).

Because chromosome congression partly rescued the end-tethering defect caused by constitutive Aurora-B activity, we tested if congression can rescue SKAP depletion induced end-tethering defect. Analyzing KT-MT attachments in bipolar spindles following MG132 and ZM447439 treatment showed significantly more lateral kinetochores in SKAP depleted cells, compared to control depleted cells (Fig. 5g and Supplementary Fig. 8D). These data show that SKAP is essential for defining the plane of KT-MT interaction in both bipolar and monopolar spindles.

## Discussion

First, we report a novel role for Aurora-B pathway in controlling the plane of KT-MT attachment in human cells. This role for Aurora-B in the maturation of kinetochore-microtubule attachments is distinct from it’s well established role in detaching erroneous KT-MT attachments (syntelic or merotelic)^23, 25, 49^. Second, we show that increasing Aurora-B at the centromere or outer-kinetochore promotes the incidence of lateral attachments by influencing different steps of the end-on conversion process. We find that lateral KT-MT attachments are ‘immune’ to Aurora-B regulation. Third, of the two Aurora-B counteracting phosphatases we tested, BubR1 associated PP2A is more important than KNL1 associated PP1 for defining the plane of KT-MT attachment, revealing functional differences between these two outer-kinetochore associated phosphatases. Finally, we uncover a role for the Astrin-SKAP complex in ensuring the lateral to end-on conversion of attachments and maintenance of end-tethered kinetochores. By comparing kinetochores in bipolar and monopolar spindles, we distinguish distinct sequential regulators of the end-on conversion process. Thus, we provide first insight into how the Aurora-B pathway controls the end-on conversion process in human cells.

Our live-cell imaging studies shed light on a physiologically significant puzzle about how human cells protect immature lateral attachments from being destabilized by the error-correction enzyme Aurora-B^50^. We find that increasing centromeric Aurora-B detaches erroneous syntelic (end-on) attachments but spares immature lateral attachments. These data from monopolar spindles show that immature lateral attachments are neither susceptible to Aurora-B’s error correction activity nor reliant on biorientation, explaining how early-on attachments could form despite a lack of inter-kinetochore tension. Similar protection of immature lateral attachments against destabilization by Aurora-B/Ipl1 is reported in budding yeast^17^.

**Fig. 6:**
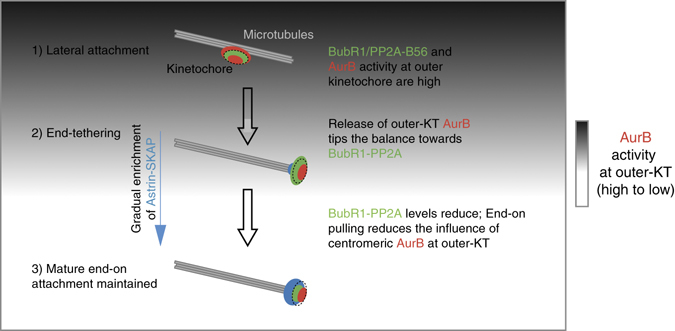
Schematic description of the end-on conversion process. On laterally attached kinetochores, outer-kinetochore associated Aurora-B kinase and BubR1-PP2A phosphatase levels are high. End-on conversion requires the reduction of outer-kinetochore associated Aurora-B activity, which potentially tips the balance in favour of BubR1-associated PP2A phosphatase allowing the gradual recruitment of Astrin-SKAP complex. Concomitantly, end-tethered kinetochores experience end-on pulling and intra-kinetochore tension, which can spatially separate centromeric Aurora-B from outer-kinetochore substrates. This progressive reduction in Aurora-B activity at the outer-kinetochore will promote further enrichment of Astrin-SKAP complex—a crucial late event in end-on conversion that is essential to maintain mature end-on attachments. *Red circles* mark outer and inner kinetochore associated Aurora-B, *green circles* mark BubR1 and *blue circles* mark Astrin-SKAP

In monopolar spindles, which closely mimic early mitotic spindle configuration, we show that Aurora-B activity at the outer-kinetochore allows lateral attachments but blocks end-on conversion. Aurora-B activity at the outer kinetochore is potentially counteracted through BubR1-PP2A that is high on early mitotic kinetochores. Loss of outer-kinetochore associated Aurora-B is likely to tip the balance towards BubR1-associated PP2A phosphatase allowing end-on conversion. Concomitantly, selective enrichment of the Astrin-SKAP complex on MT-end tethered kinetochores^4^, along with the physical separation of centromeric Aurora-B from outer-kinetochore substrates by end-on attachment mediated kinetochore pulling, could jointly facilitate the maintenance of a mature end-on attachment (Fig. 6).

Previous studies showed that Astrin is not recruited to uncongressed kinetochores lacking BubR1 associated PP2A^48^. Whether this failure to recruit Astrin is simply due to the lack of congression or directly due to the loss of PP2A was not known. We demonstrate that both Astrin recruitment and end-on attachment status are compromised on congressed kinetochores lacking BubR1 associated PP2A. Thus, BubR1 associated PP2A is required for proper KT-MT attachment even on congressed chromosomes, indicating a new role for BubR1 that is present on metaphase kinetochores^51^. Whether the lack of BubR1-PP2A mediated end-on conversion event(s) is an important reason for higher sensitivity to loss of BubR1 in Brain Tumour Initiating Cells compared to normal cells^52^ would be interesting to explore in the future.

How does the Astrin-SKAP complex mediate end-on conversion? Electron microscopy studies indicate that SKAP^159–316^: Astrin^482–850^ complex form elongated structures^53^, and Astrin dimers show a ‘lollypop’ like structure of 80 nm in length with a flexible hinge^54^. We and others previously showed that a similar hinge region in Ndc80 (loop domain) is required for tethering kinetochores onto microtubule-ends^4, 11–13^. Unlike Ndc80, which is a core kinetochore protein, the Astrin-SKAP complex is recruited to kinetochores at later stages of mitosis^55–57^ and found predominantly on end-tethered kinetochores in human cells^4^. Future structure studies on Astrin’s hinge domain and the kinetochore receptor(s) of Astrin-SKAP complex are likely to reveal crucial insights into how human cells overcome physical barriers in converting the plane of kinetochore-microtubule attachment. Although Astrin-SKAP complex is essential for normal chromosome segregation in human cells^41, 55–58^, a truncation mutation in exon-3 of SKAP did not cause somatic defects in mice^59^, suggesting that redundant players may support the loss of SKAP in mice.

In summary, our findings uncover Aurora-B kinase as an upstream regulator of the end-on conversion process. Unlike spindle checkpoint mechanisms that rely on the two phosphatases KNL1-associated PP1 and BubR1-associated PP2A-B56, the end-on conversion process relies significantly on BubR1-associated PP2A-B56. Finally, we identify a role for Astrin-SKAP in a late event of the end-on conversion process. Thus, we report one of the key molecular pathways that control the plane of kinetochore-microtubule interaction in human cells.

## Methods

### Cell culture and synchronization

HeLa cells (ATCC) cultured in Dulbecco’s Modified Eagle’s Media supplemented with 10% FCS and antibiotics (Penicillin and Streptomycin) were plated onto glass-bottomed dishes (LabTek) or 13 mm round coverslips. For inhibition studies, cells were treated with 10 µM Monastrol (1305, TOCRIS), 10 µM ZM447439 (2458, TOCRIS) or 10 µM MG132 (1748, TOCRIS). Inducible expression of GFP-SKAP^siRes^ was achieved by generating HeLa (GFP-SKAP) as clones obtained from transfecting HeLa FRT/TO cells with pCDNA5-FRT/TO-GFP-SKAP^siRes^ plasmid vector^41^.

### Plasmid and siRNA transfections

siRNA transfection was performed using Oligofectamine according to manufacturers instructions. Briefly, siRNA oligos were incubated in OptiMEM (Invitrogen; 11058-021) and washed 24–36 h prior to analysis. siRNA oligos to deplete Astrin (UCCCGACAACUCACAGAGAAAUU), SKAP (GAAAGAGUCCGAUUCCUAGUU), BubR1 (GAUGGUGAAUUGUGGAAUA) and KNL1 (GCAUGUAUCUCUUAAGGAAUU) were from Dharmacon. Negative control siRNA (12,935–300) was from Invitrogen. HeLa^YFP-Tub; CENPB-Red^ was generated by transiently transfecting pDsRed-CENPB (Clonetech) into HeLa^YFP-Tub^ used extensively for cell division studies^60, 61^. In four well LabTek dishes, 500 ng of plasmid vectors encoding mKate2-αTubulin (Evrogen) along with plasmid vector encoding Mis12-INCENP-GFP (VSV-Mis12-ΔCen INCENP-GFP), Mis12-INCENP(TAA)-GFP (VSV-Mis12-ΔCen INCENP(TAA)-GFP), CenpB-INCENP-GFP (VSV-CenpB-ΔCen INCENP-GFP) or CenpB-INCENP(TAA)-GFP (VSV-CenpB-ΔCen INCENP(TAA)-GFP)^26^ were co-transfected into HeLa using Turbofect (R0531, Thermo Scientific) and filmed within 30 h. LAP-tagged KNL1 expression vectors^4, 37^ were transiently transfected prior to Doxycycline induction. Following transient transfection of plasmids, cells were assessed after 24 h, except in the case of KNL1 expression plasmids where cells were assessed after 30 h. For SKAP siRNA rescue, Tetracycline (Tet) inducible GFP-SKAP HeLa cells were transfected with SKAP siRNA^43^, 48 h later treated with tetracycline for 1 and 24 h later used for studies. siRNA resistant version of GFP-SKAP expression vector (pCDNA5-FRT/TO-GFP-SKAP^siRes^) was generated by wobbling nucleotides using point mutagenesis primer GTAAAGCTGGAGATGAAAGAGGAGCGTGTTAGGT TTTTGGAACAGCAAACCTTATG. Plasmid vectors maps are available on request.

### Live-cell time-lapse imaging

Cells were transfected with siRNA oligos or plasmid vectors 72 or 24 h, respectively, prior to imaging (unless stated otherwise in experimental regime) and transferred to Leibovitz’s L15 medium (Invitrogen; 11415064) for imaging at 37 °C. For imaging KT and MT-end dynamics, exposures of 0.04 s and at least 6 *Z*-planes, 0.1 μm apart, were acquired every 10 s for 5 min using a 100X NA 1.40 oil immersion objective on an Applied Precision DeltaVision Core microscope equipped with a Cascade2 camera under EM mode. Exposure conditions were optimized through long-term high-speed imaging of mKate2-EB3 expressing HeLa cell line^62, 63^.

### Kinetochore-microtubule attachment status determination

To define KT-MT attachment states, we used the following methodology. First, 360° rotation of image stacks (3D volume images) generated using Softworx was used to determine the plane of the K-fibre and KT. Second, end-tethered and lateral kinetochores were confirmed on the basis of their synchronous and asynchronous movement, respectively, between the K-fibre tip and the KT. Change in KT and MT-end (MT) positions through time was measured using Softworx distance measurement tool. Values less than 0.25 μm, sustained for at least 30 s, were marked as synchronous movement of the KT and MT-end. All other values were marked as asynchronous movement of the KT and MT-end. To ensure that the attachment transition measurements are reliable, we only considered end-on kinetochores that remained in end-on state at least for 30 s, lateral kinetochores that remained lateral for greater than 30 s and detached kinetochores that remained detached for at least 20 s. (In Mis12-INCENP-GFP and mKate2-Tubulin coexpressing cells, we expect a signal overlap since Mis12 resides in the KT outer plate where MT-ends reach^64^. However, in CenpB-DsRed and YFP-Tubulin coexpressing cells, signal overlap is rarely seen). In fixed-cells to set a threshold for high-quality images for quantitative analysis, we excluded cells if greater than 20% of its kinetochores could not be confidently assigned any KT-MT attachment status. Lateral, end-on or detached kinetochores were scored^4^ and their relative percentages calculated using *MS Excel* and plotted using *Prism* Software.

### Immunofluorescence, immunoblotting and image analysis

For immunofluorescence, antibodies against HEC1^Ndc80^ protein^65^ (1:1000), tubulin (Abcam; ab6160; 1:800), pThr232 Aurora-B (Rockland Inc; 600-401-677; 1:500), total Aurora-B (Abcam; ab3635; 1:800), GFP (Roche; 1181446001; 1:800), mCherry (Thermo Scientific; M11217; 1:2000), SKAP (Atlas; HPA042027; 1:1000), HEC1pSer44 and HEC1pSer55^28^ (1:500), Astrin (Novus; NB100-74638; 1:1000), B56 (BD Transduction Laboratories; 610615; 1:500) and CREST antisera (Europa; FZ90C-CS1058; 1:2000) were used. DAPI (Sigma) was used to co-stain DNA. Images of immunostained cells were acquired using 100X NA 1.4 objective on a DeltaVision Core microscope equipped with CoolSnap HQ Camera (Photometrics). Volume rendering (*SoftWorx*) was performed for 3D analysis of KT-MT attachment status. Deconvolution of live and fixed-cell images and 3D volume rendering were performed using SoftWorx. Quantitative immunoblotting was performed on proteins separated on 12% SDS-PAGE gels by transferring them overnight onto Nitrocellulose membrane. Membranes were incubated in primary antibodies against Aurora-B pThr232 (Rockland Inc; 600-401-677; 1:1000), SKAP (Atlas; HPA042027; 1:1000) and γ-Tubulin (Sigma; T6557; 1:800) for an hour and probed using secondary antibodies labeled with infrared fluorescent dyes, which were imaged using an Odyssey imager (Supplementary Fig. 9).

### Statistical analysis

To confirm that sampling is sufficient, we used two statistical tests: First, we performed a proportion test or unpaired Student’s *t*-test to confirm using *P*-values that we have sampled sufficient number of cells or kinetochores for concluding on differences we report. Second, we measured standard error over mean (SEM) across experimental repeats or across cells and confirmed that the differences we report are not only based on differences in mean values, but also the spread of the mean values between experiments, cells or kinetochores. SD values were obtained across experiments or cells as indicated in the figure legend.

### Super-resolution microscopy experiments

For STORM imaging experiments, HeLa cells were transiently transfected with plasmids encoding Venus-BubR1 ∆660–685 and 24 h later cells were exposed to MG132 for 1 h. Cells were then pre-fixed with 4% PFA in PBS for 20 s, permeabilised with 0.5% Triton X-100 in PBS for 4 min and the fixation step was repeated again for 20 min before quenching with 25 mM glycine for 20 min. One percent BSA in PBS with 0.1% Tween was used for blocking and all washes were performed using 0.1% tween in PBS. Cells were immunostained with primary antibodies against α-Tubulin (clone B-5-1-2, Sigma-Aldrich; T6074; 1:300) and CREST antisera (Europa; FZ90C-CS1058; 1:2000). For secondary antibody, goat anti-mouse AlexaFluor 647 (Molecular Probes; A21236; 1:800) and donkey anti-human CF680 (Biotium; 20278–1; 1:1000) were used.

Samples were mounted and imaged in a custom-made microscope^66^ and covered with 300 µl of imaging buffer (150 mM Tris-HCl pH 8, 10% (v/w) glucose, 35 mM cysteamine (MEA), 0.5 mg/ml glucose oxidase (Sigma; G7141), and 40 mg/ml catalase (Sigma; C3556)). Typically, 200,000–500,000 frames were recorded. Analysis was performed using custom software written in MATLAB. We acquired two spectral channels simultaneously by splitting the emission with a dichroic (Chroma, T680LPXXR) and assigned the colour based on the relative intensities of the single-molecule localizations in both channels with a cross-talk rate below 0.2%^67^. Localizations with uncertainties above 25 nm or a fitted size of the PSF above 170 nm were discarded. The data were corrected for sample drift using a custom redundant cross-correlation based algorithm. Images were rendered using a Gaussian with a width proportional to the localization precision.

### Data availability

The data that support the findings of this study are available from the corresponding author upon reasonable request.

## Electronic supplementary material

Supplementary Information
