# Supplementary material for: Aurora-B kinase pathway controls the lateral to end-on conversion of kinetochore-microtubule attachments in human cells
Source: Nat Commun. 2017 Jul 28;8:150. doi: 10.1038/s41467-017-00209-z (PMC5532248; doi:10.1038/s41467-017-00209-z)
Supplement: Supplementary file 1 — Supplementary Information [file 41467_2017_209_MOESM1_ESM.pdf]

Title: Supplementary Information

Description: Supplementary Figures

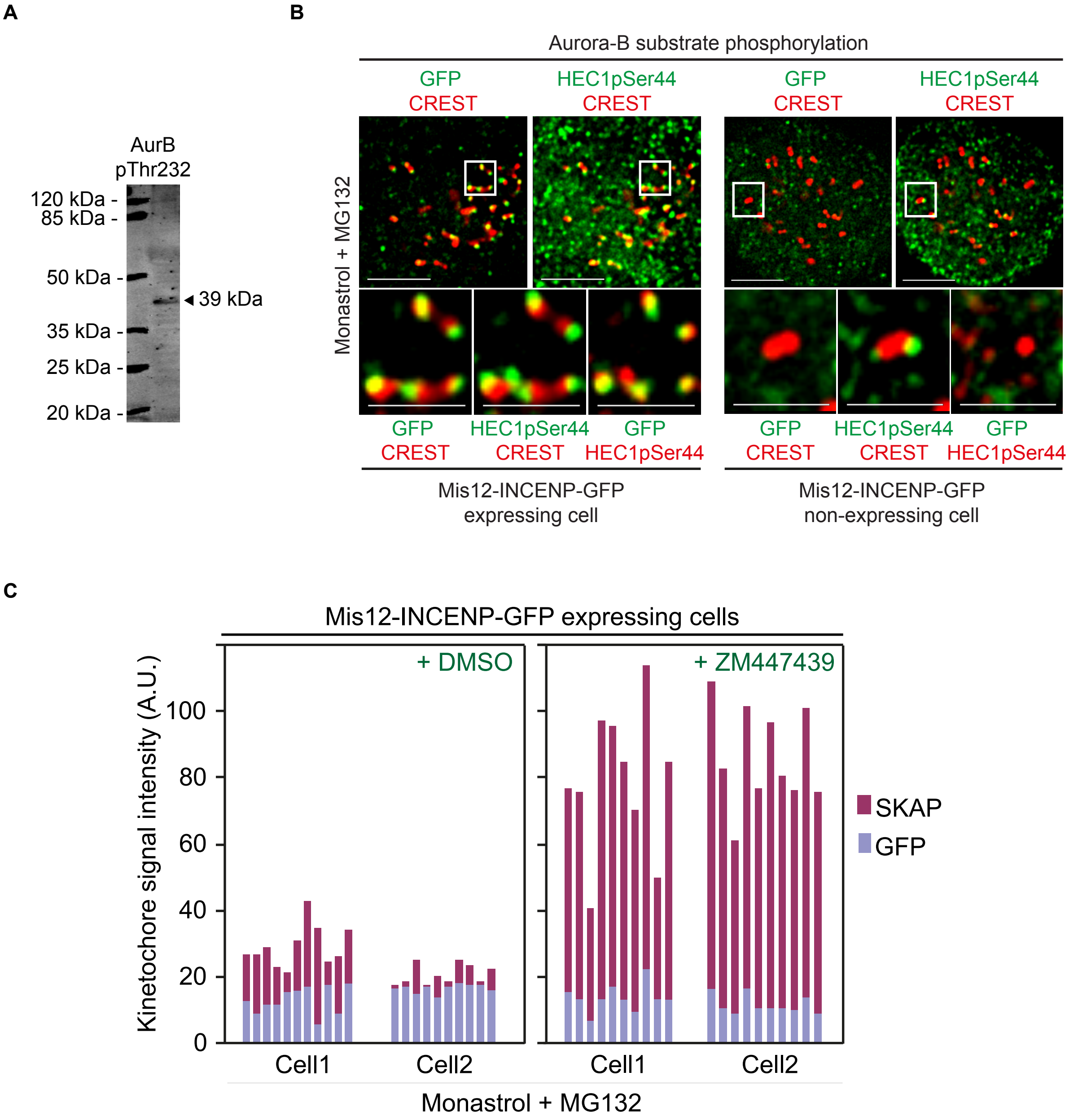

Supplementary Figure 1: Aurora-B signaling mediated changes in levels of kinetochore protein and phosphorylation.

(**A**) Immunoblot of lysates of HeLa cells showing the specificity of Aurora-BpThr232 antibody (expected molecular weight: 39 kDa). (**B**) Images showing the extent of HEC1pSer44 signals in kinetochores of Mis12-INCENP-GFP expressing and non-expressing cells. Cells were treated with Monastrol and MG132 and immunostained with antibodies against HEC1pSer44, GFP and CREST antisera. Scale bars: 5 μm in uncropped and 2 μm in cropped images. Boxed areas correspond to cropped images. (**C**) Graphs show intensity of KT bound SKAP in cells expressing Mis12-INCENP-GFP treated as in Figure 1D.

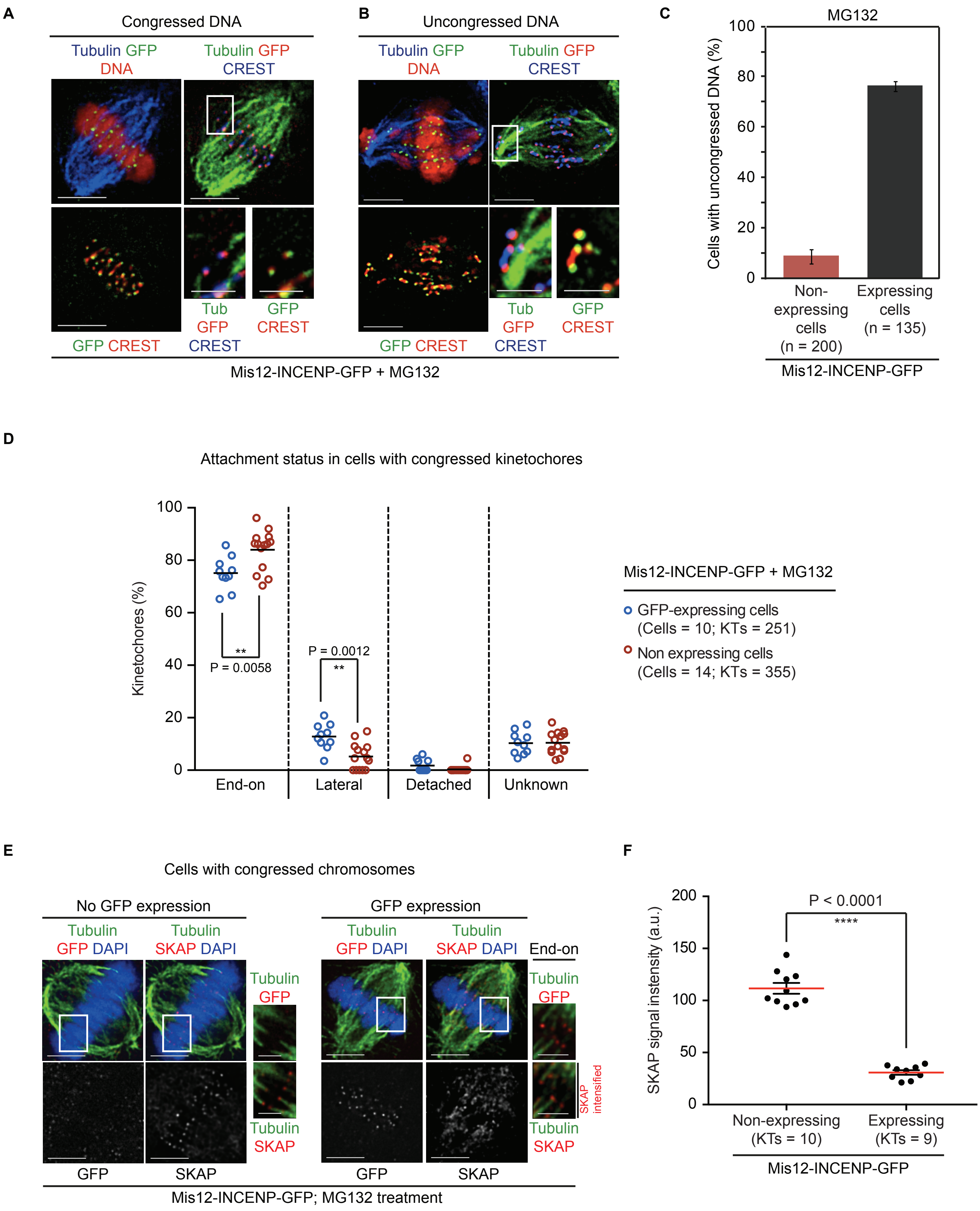

Supplementary Figure 2

Supplementary Figure 2

Chromosome congression partially resolves lateral attachments induced by constitutive Aurora-B at the outer-kinetochore

(**A** and **B**) Images of cells show congressed end-tethered kinetochores (**A**) or uncongressed lateral kinetochore pairs (**B**). Cells transfected with Mis12-INCENP-GFP expression vector were exposed to MG132 prior to immunostaining with antibodies against Tubulin (Tub) and GFP and CREST antisera. DNA was costained using DAPI. (**C**) Graph shows percentage of Mis12-INCENP-GFP expressing or non-expressing mitotic cells with uncongressed DNA following treatment as in A and B. Error bar represents SEM across two experiments. (**D**) Graph showing KT-MT attachment status in congressed chromosomes of Mis12-INCENP-GFP expressing cells as in A. Each circle represents data values from one cell. Horizontal lines show average values across cells from two independent experimental repeats. (**E** and **F**) Images (**E**) and intensity graph (**F**) show KT-bound SKAP in cells with or without Mis12-INCENP-GFP expression exposed to MG132 prior to immunostaining with antibodies against Tubulin, GFP and SKAP. Red and black bars represent average and SD values across kinetochores, respectively. In D and F, '*' refer to significant differences, based on P-values derived using unpaired Student's t-test from two independent experimental repeats. In A, B and E, boxed areas correspond to cropped images; Scale bar: 5 μm in uncropped and 2 μm in cropped images.

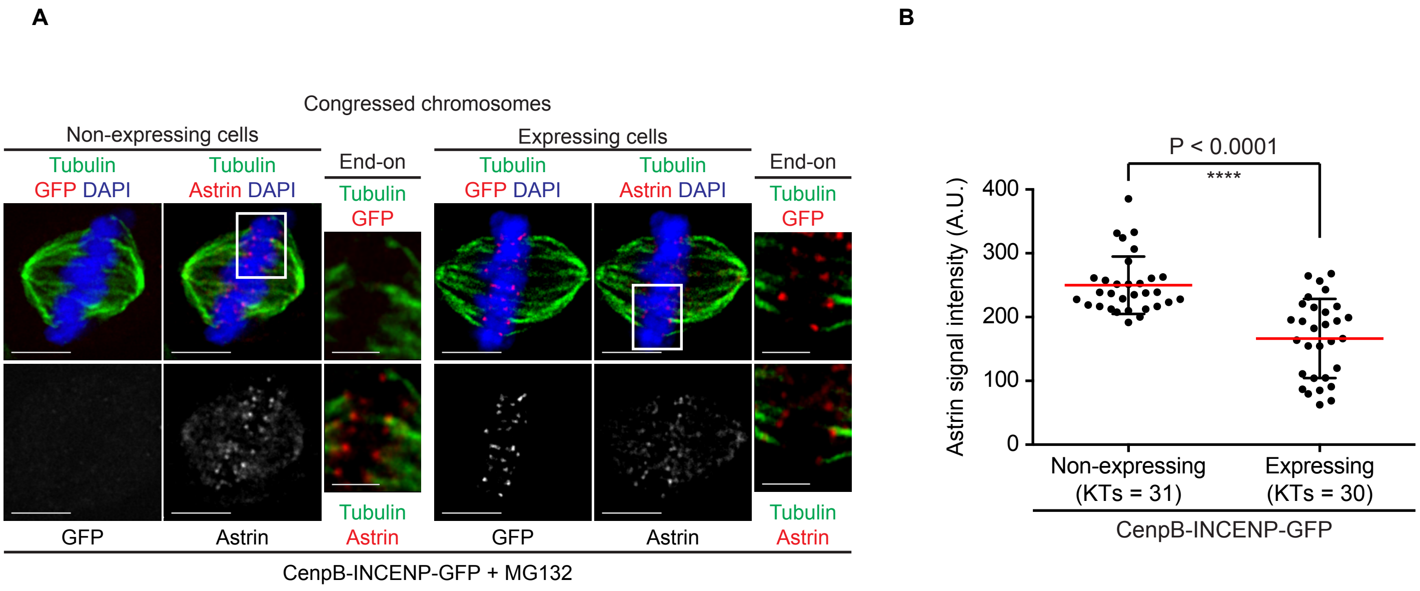

Supplementary Figure 3

Astrin recruitment extent in cells with Aurora-B activity at the centromere

(**A** and **B**) Images (**A**) and intensity graph (**B**) of KT bound Astrin in CenpB-INCENP-GFP expressing or non-expressing cells exposed to MG132 and immunostained with antibodies against GFP, Tubulin and Astrin and stained with DAPI for DNA. Scale bar: 5 μm in uncropped and 2 μm in cropped images. '*' refer to significant differences based on P-values derived using unpaired Student's t-test from two independent experimental repeats. Red and black bars represent average and SD values, respectively, across kinetochores.

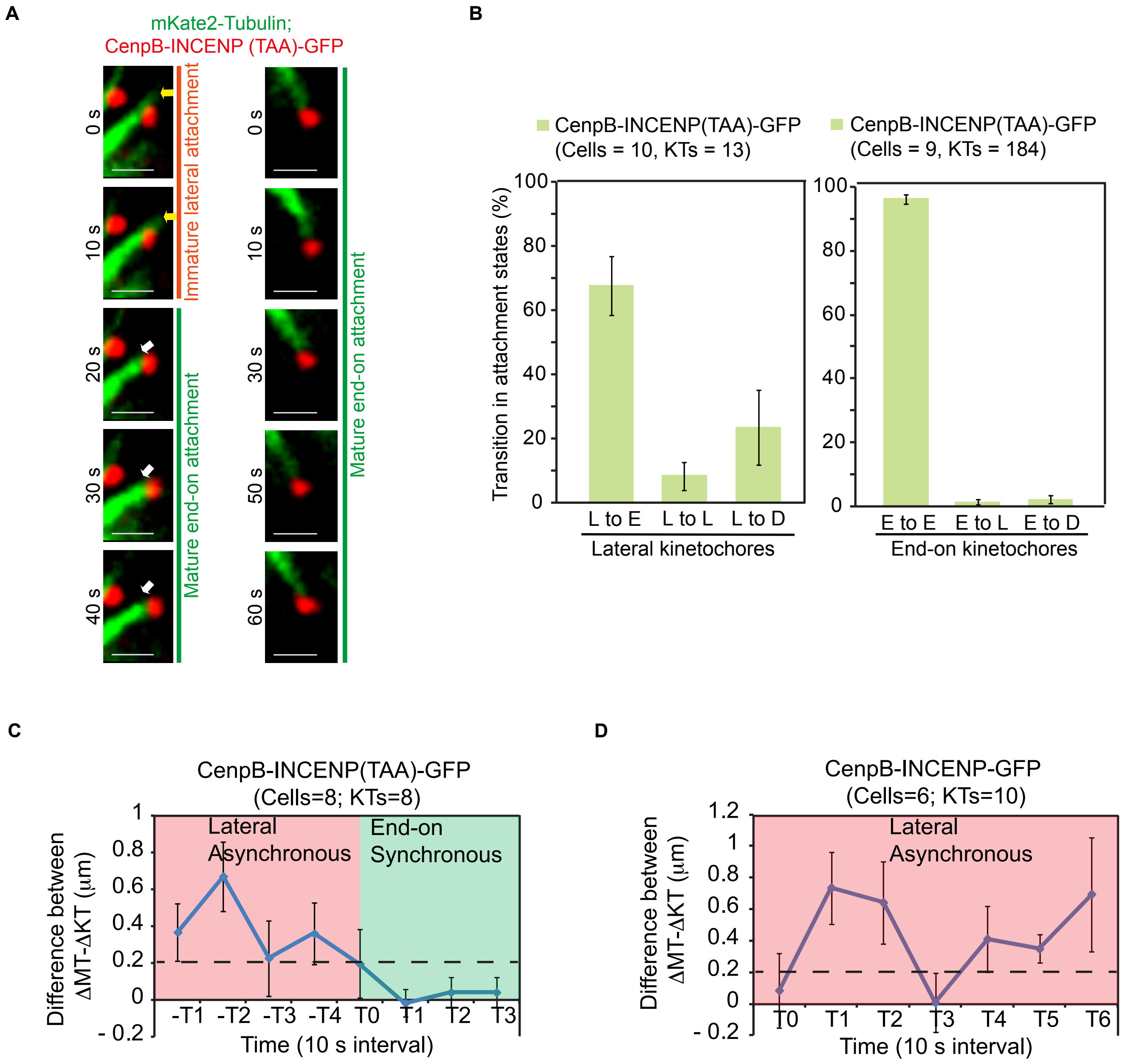

Supplementary Figure 4: Fate of lateral kinetochores in cells with Aurora-B at the centromere (**A**) Single-plane time-lapse images of Z-stacks show the fate of a lateral or end-on KT (red) bound to MT (green) in cells co-expressing mKate2-Tubulin and CenpB-INCENP(TAA)-GFP exposed to Monastrol. Yellow arrows mark tip of lateral K-fibre. White arrows highlight synchronous movement of the KT and MT-end. Scale bar: 2 μm. (**B**) Graph shows percentage of lateral or end-on kinetochores that transitioned into other attachment states (D-detached, L-lateral and E-end-on) in time-lapse images as in A. Error bars are SEM values across three experimental repeats. (**C**) Graph shows the difference between ΔMT (change in MT-end position) and ΔKT (change in KT position) through time in CenpB-INCENP(TAA)-GFP and mKate2-Tubulin co-expressing cells. Values were obtained from time-lapse images as in A. Values less than 0.25 μm (marked by dashed line), sustained for at least 30 s, are indicated as synchronous movement (green) of the KT and MT-end. All other values are indicated as asynchronous movements (pink) of the KT and MT-end. (**D**) Graph shows the difference between change in KT and MT-end (MT) positions through time in CenpB-INCENP-GFP and mKate2-Tubulin co-expressing cells as in Figure 3C. Values less than 0.25 μm (dashed line) not sustained for at least 30 s or values more than 0.25 μm indicated as asynchronous movements between the lateral KT and MT-end.

A

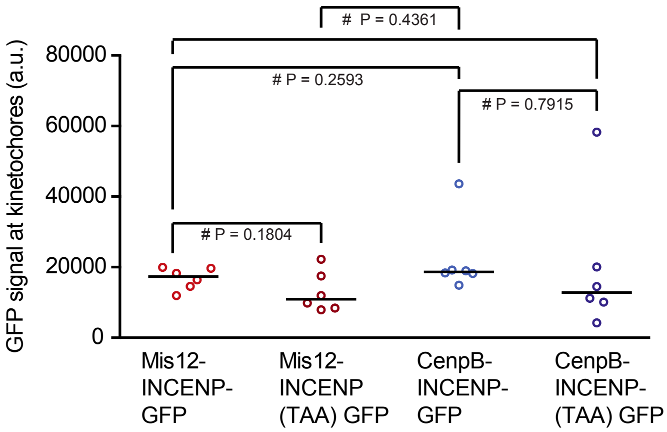

B

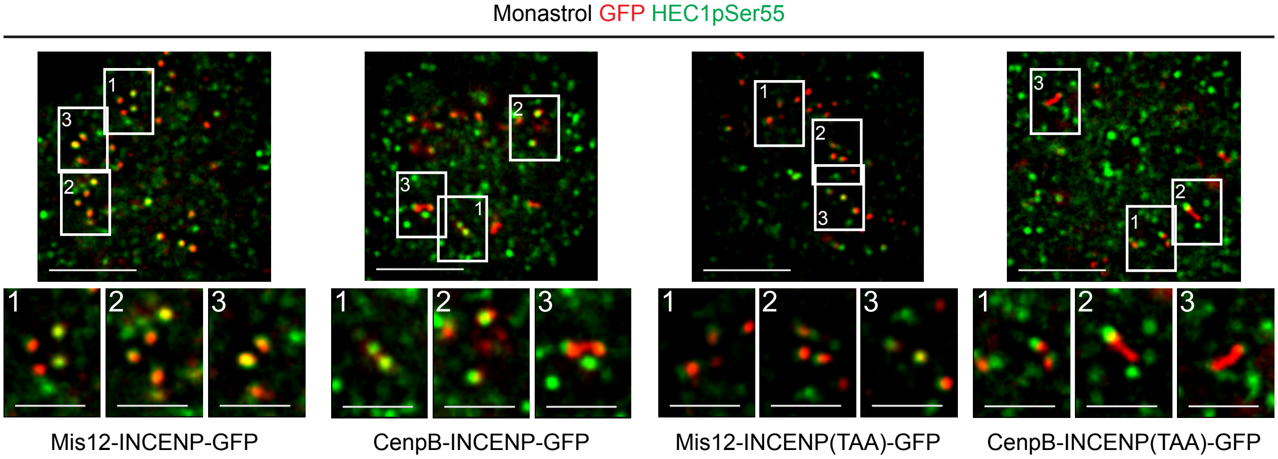

C

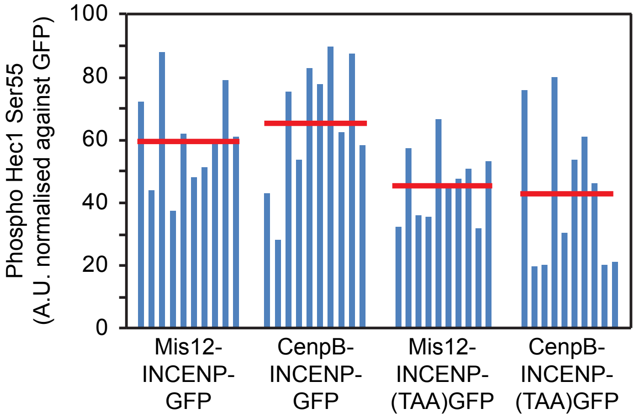

Supplementary Figure 5

Supplementary Figure 5

Phosphorylation extent of outer kinetochore protein HEC1, as a means to measure Aurora-B activity at the outer kinetochore, in cells expressing various INCENP-GFP fusions

(A) Graph shows average intensity of KT-bound GFP in monastrol-treated live-cells expressing mKate2-Tubulin and one of the GFP fusions, as indicated: Mis12-INCENP-GFP, CENPB-INCENP-GFP, Mis12-INCENP(TAA)-GFP or CenpB-INCENP(TAA)-GFP. Horizontal bars indicate average intensity values across cells. '#' indicates statistically insignificant differences based on P-values derived using unpaired Student's t-test from three independent experimental repeats. (B and C) Immunofluorescence images (B) and graph (C) show the intensity of KT bound HEC1pSer55 in cells expressing fusion proteins as indicated: Mis12-INCENP-GFP, CENPB-INCENP-GFP, Mis12-INCENP(TAA)-GFP or CenpB-INCENP(TAA)-GFP. Cells were treated with Monastrol before immunostaining with antibodies against GFP and HEC1pSer55 and costained with DAPI for DNA (DNA signal not shown). Insets correspond to areas marked with white boxes. Scale bar: 5 μm in uncropped and 2 μm in cropped images. Red bars indicate average intensity values across kinetochores.

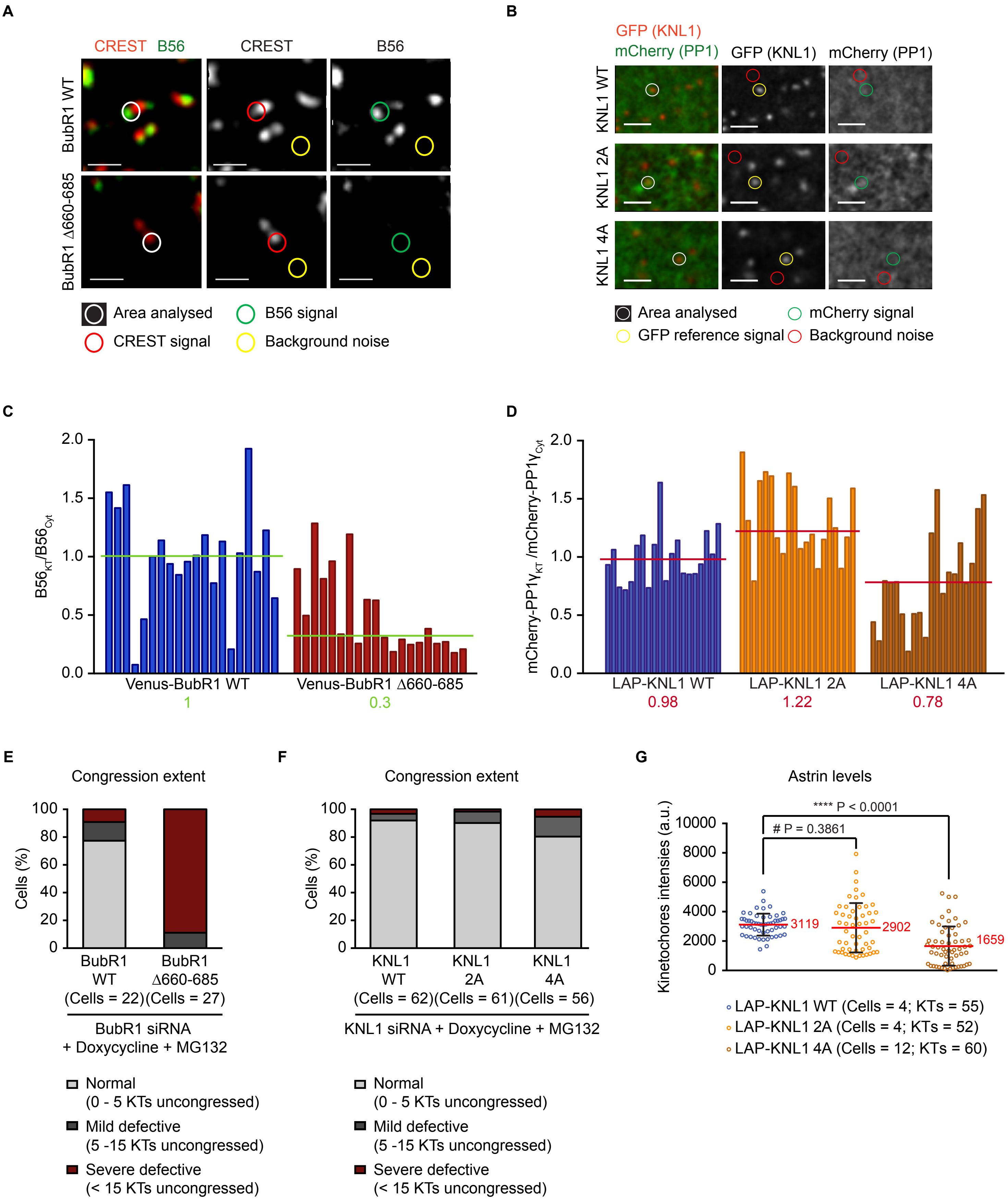

Supplementary Figure 6

Supplementary Figure 6

Levels of PP2A and PP1 phosphatases are reduced on kinetochores of cells expressing BubR1 Δ660-885 and KNL1 4A mutants, respectively.

(**A** and **B**) Images of kinetochores show circled areas used to measure intensities of PP2A-B56 (A) or mCherry-PP1 (B), pseudo-coloured in green, in cells depleted of either BubR1 (A) or KNL1 (B) and conditionally expressing wild type or mutant forms of BubR1 (A) or KNL1 (B) as shown. Scale bar: 1μm. Cells were treated with Monastrol (A) or MG132 (B) before immunostaining with antibodies against GFP and either PP2A-B56 (A) or mCherry (B) for mCherry-PP1γ fusion protein. Average background noise from 6 random points outside the cell was measured and subtracted from individual kinetochore and cytoplasmic intensities. For cytoplasmic value, an average of 6 random points within the same cell was used. (**C** and **D**) Graphs showing intensity ratios of KT bound PP2A-B56 (C) or PP1 (D) relative to cytoplasmic intensities in cells treated as in (A) and (B), respectively. Each vertical bar represents a kinetochore intensity value normalized against the average intensity value in wild-type control cells. Horizontal bars and numerical values (in red or green) refer to median values across kinetochores from two independent experimental repeats. (**E** and **F**) Graphs show the extent of chromosome congression defect in MG132 treated cells depleted of either BubR1 (E) or KNL1 (F) and conditionally expressing wild type or mutant forms of BubR1 or KNL1, respectively, in the presence of Doxycycline. Extent of alignment defect was measured using Tubulin, kinetochore and DNA signals in immunostained cells as in Figure 4D and Figure 4F. Data was obtained from at least three independent repeats. (**G**) Graph showing the intensity of kinetochore-bound Astrin in KNL1 depleted cells expressing LAP-tagged KNL1 WT or mutants (4A or 2A). Cells were treated as in Figure 4F. Each circle represents intensity value from one kinetochore. Horizontal bar and numerical values (in red) are average intensity values across KTs from three independent experimental repeats. '*' and '#' indicate statistically significance and insignificant differences, respectively (assessed using P-values from unpaired Student's t-test).

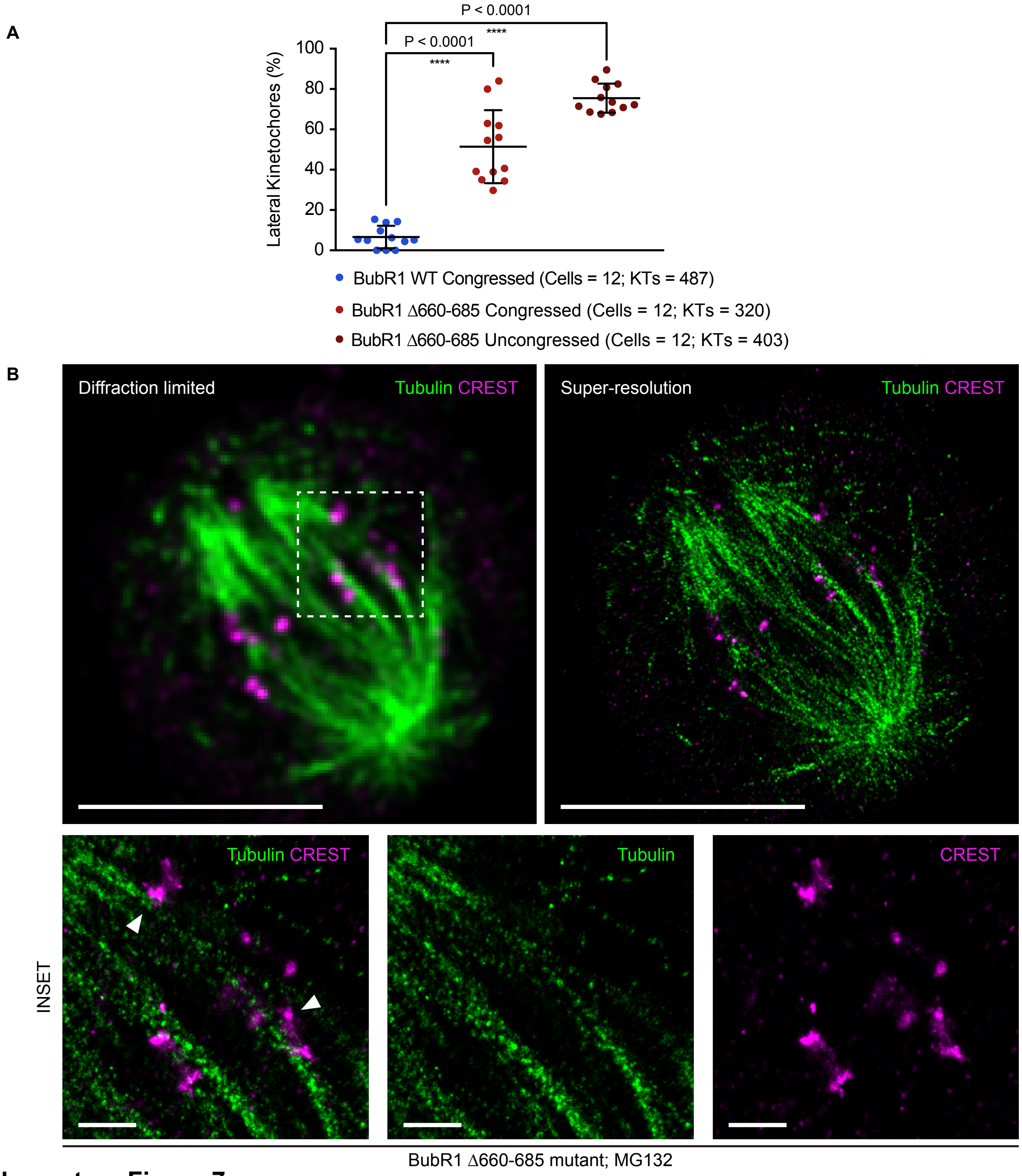

Supplementary Figure 7

Congressed kinetochores are laterally attached in cells expressing BubR1 Δ660-685 mutant

(**A**) Graph showing the percentage of lateral KT-MT attachments in congressed and uncongressed chromosomes of BubR1-depleted cells conditionally expressing either Venus-BubR1 WT or Δ660-685 mutant following Doxycycline induction and MG132 treatment. Each circle represents one cell. Black horizontal bar and whiskers mark average values and standard deviation, respectively, from three independent experiments. '*' indicates statistically significant differences (assessed using P-values from unpaired Student's t-test). (**B**) Diffraction limited and reconstructed super-resolution images, as indicated, of a representative cell expressing Venus-BubR1 Δ660-685 mutant acquired using dual-colour STORM (n(cells)=4; KT-MT attachment status: 33 lateral; 9 end-on; 18 undetermined). Cells were arrested in metaphase for 1 h using MG132 prior to fixation and immunostaining with CREST antisera and α-Tubulin antibody. Venus signal (not shown) was used to identify mutant expressing cells. Insets are cropped super-resolution images corresponding to area marked using dashed lines. White triangles mark lateral kinetochores. Scale: 10 μm in uncropped and 1 μm in cropped images.

A

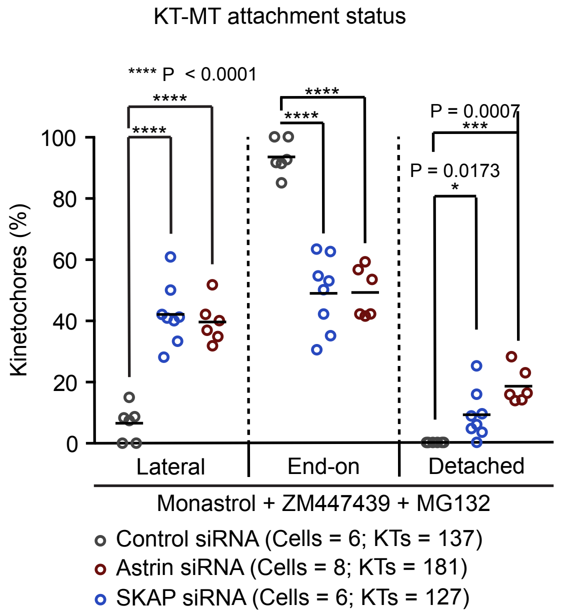

B

Experimental Regime

siRNA treatment → 48 h → Tet induction → 1 h → Wash Tet, add media without Tet → 20 h → Monastrol → 2 h → ZM447439 +MG132 → 45 min → Immunostaining

C

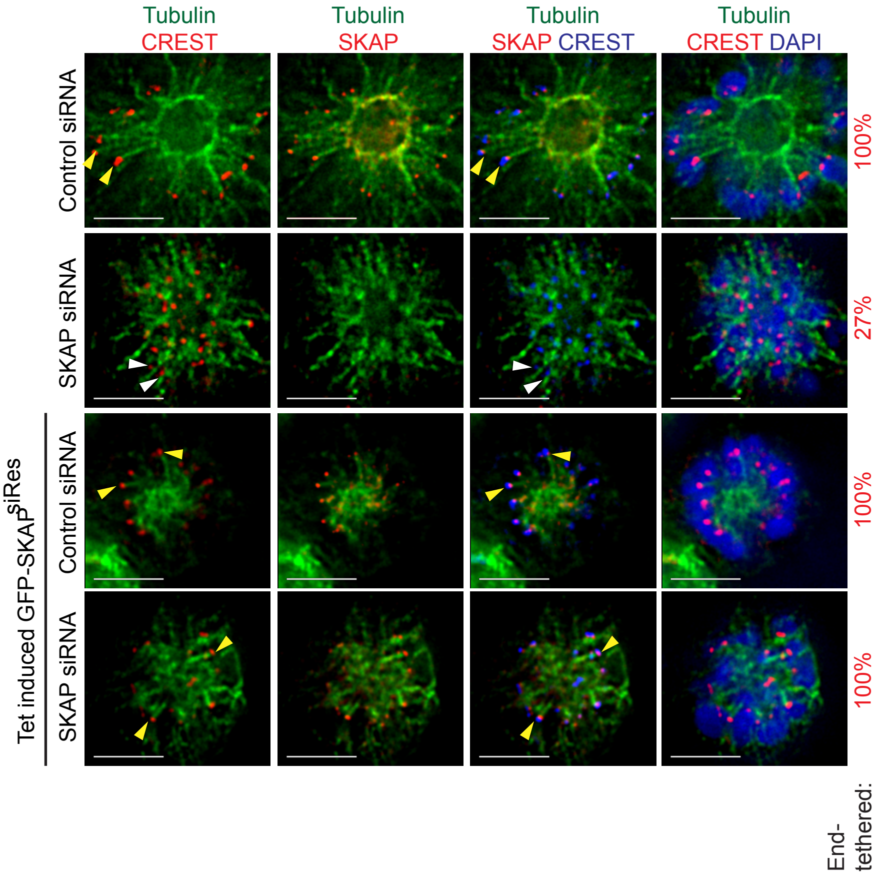

D

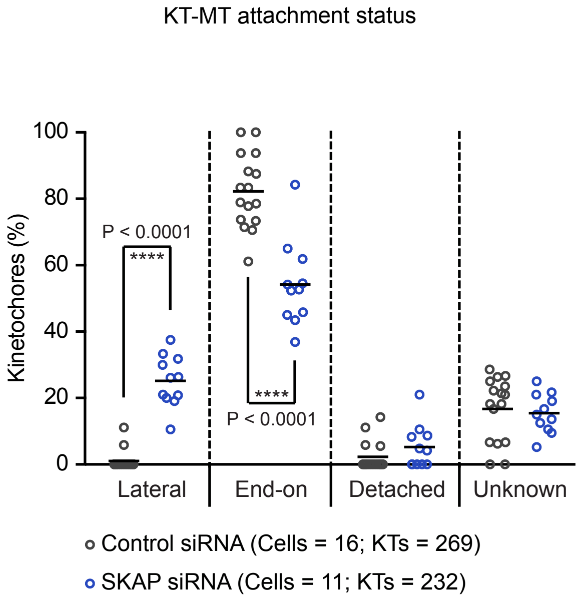

Supplementary Figure 8

Supplementary Figure 8

GFP-SKAP (siRes) rescues the end-tethering failure caused by SKAP siRNA treatment

(**A**) Graph shows percentage of lateral, end-on or detached kinetochores in monopolar spindles of siRNA treated cells as in Figure 5B. Each circle represents value from one cell. Black horizontal bar marks average values. (**B**) Schematic describing experimental methodology for C: siRNA treated HeLa or HeLa (GFP-SKAPsiRes) cells were exposed to Tetracycline (Tet) briefly and then released to Tetracycline-free media as indicated. Cells were treated with Monastrol for 2 h and either ZM447439 or DMSO along with MG132 for 45 min prior to immunostaining with antibodies against Tubulin and SKAP, CREST antisera and stained with DAPI for DNA. (**C**) Immunofluorescence images of cells transfected with control or SKAP siRNA and treated as indicated in B. Yellow and white arrows show end-on and lateral kinetochores, respectively. Scale bar: 5 μm. Percentage values in (red) refer to cells with predominantly end-tethered kinetochores that were positioned uniformly distant from spindle poles. (**D**) Graph shows percentage of lateral, end-on or detached kinetochores in bipolar spindles of siRNA treated cells, assessed from images as in Figure 5G. Each circle represents value from one cell. Black horizontal bar marks average values. '*' refers to significant difference based on P-value derived using Unpaired Student's t-test from two (D) or three (A) independent experiments.

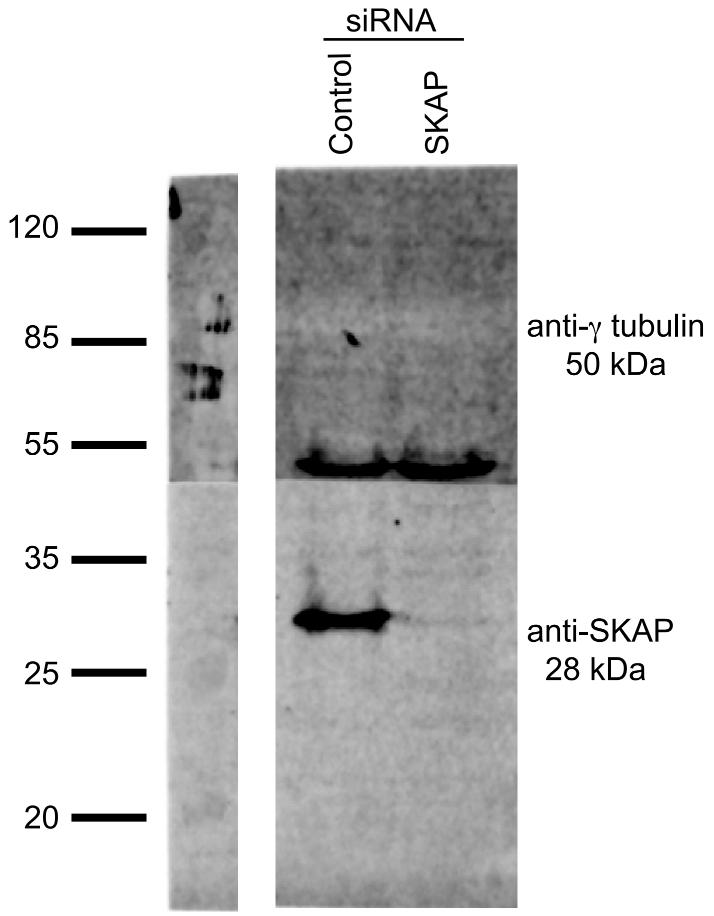

Supplementary Figure 9: Uncropped scans of immunoblots related to images presented in main Figure 5F demonstrating depletion of SKAP in cells treated with SKAP siRNA.
